# Impact of e-resources on learning in biochemistry: first-year medical students’ perceptions

**DOI:** 10.1186/1472-6920-12-21

**Published:** 2012-05-16

**Authors:** Joe Varghese, Minnie Faith, Molly Jacob

**Affiliations:** 1 Department of Biochemistry, Christian Medical College, Vellore, 632002, India

## Abstract

**Background:**

E-learning resources (e-resources) have been widely used to facilitate self-directed learning among medical students. The Department of Biochemistry at Christian Medical College (CMC), Vellore, India, has made available e-resources to first-year medical students to supplement conventional lecture-based teaching in the subject. This study was designed to assess students’ perceptions of the impact of these e-resources on various aspects of their learning in biochemistry.

**Methods:**

Sixty first-year medical students were the subjects of this study. At the end of the one-year course in biochemistry, the students were administered a questionnaire that asked them to assess the impact of the e-resources on various aspects of their learning in biochemistry.

**Results:**

Ninety-eight percent of students had used the e-resources provided to varying extents. Most of them found the e-resources provided useful and of a high quality. The majority of them used these resources to prepare for periodic formative and final summative assessments in the course. The use of these resources increased steadily as the academic year progressed. Students said that the extent to which they understood the subject (83%) and their ability to answer questions in assessments (86%) had improved as a result of using these resources. They also said that they found biochemistry interesting (73%) and felt motivated to study the subject (59%).

**Conclusions:**

We found that first-year medical students extensively used the e-resources in biochemistry that were provided. They perceived that these resources had made a positive impact on various aspects of their learning in biochemistry. We conclude that e-resources are a useful supplement to conventional lecture-based teaching in the medical curriculum.

## Background

Conventional lecture-based teaching plays a major role in teaching biochemistry in the first year of the undergraduate medical course in India. There are several reasons for this. The biochemistry curriculum is vast, the time available for teaching the subject is limited and the number of students in a class is usually large. Under such circumstances, lectures are often considered the best way to deliver considerable amounts of information to a diverse and large group of students [1-3]. However, there are many disadvantages inherent in a teaching system that is largely dependent on lectures. Delivery of a lecture by a teacher does not actively engage the learners. Students are usually passive participants in the process and often find it difficult to sustain interest in the subject over the duration of the lecture. A lecture delivered at a particular pace does not cater to all students in a class, as it is typically composed of individuals of diverse abilities. Academically weak students may find it difficult to keep pace with the lecturer as the lecture progresses. Hence, not all students are able to assimilate the content of a lecture to the same extent. Opportunities to ask questions are also limited in such a situation. In addition, the quality of a lecture primarily depends on the experience and skill of the teacher, which can be quite variable [4].

In view of the limitations associated with lecture-based teaching, which is nonetheless essential, it is imperative that medical teachers explore ways to overcome many of its inherent disadvantages. Over the last several years, the internet has had a growing influence on education. Electronic resources are now widely acknowledged as an excellent means to support learning [5,6]. Many leading universities have developed electronic learning (e-learning) material to supplement and enhance teaching.

Christian Medical College (CMC), Vellore, India, has, in collaboration, with Tufts University School of Medicine, Boston, Massachusetts, USA, developed e-learning tools in medical education. As part of this venture, CMC began to use the Tufts University Sciences Knowledge Base (TUSK) open source software platform, which was developed by Tufts University Hirsh Health Sciences Library, with the support of its medical, dental, and veterinary schools. TUSK is an enterprise-level, curriculum and multimedia knowledge management system to help faculty and students, in teaching and learning respectively [7]. The faculty of CMC has developed electronic resources to meet the requirements of teaching programs at CMC, using this system. These resources are now regularly used in the teaching programs of the institution and are available at the CMC e-learning website, which can be accessed through the URL: http://e-learning.cmcvellore.ac.in/.

The Department of Biochemistry at CMC was one of the first departments in the institution that began to develop e-resources for use by its students. The department uses the website extensively to enhance teaching and learning at undergraduate and postgraduate levels in the institution. The material that is available on the website includes (1) general information for first-year medical students on the course in biochemistry, with regard to objectives, curriculum, lecture and test schedules, assessment policies and examinations, (2) lecture presentations (PowerPoint slides), (3) a case-based tool for self-assessment (with model answers provided), (4) study material for practical classes and (5) lecture notes on the biochemical basis of diseases. In addition, the department hosts a forum on the website in which recently published research articles in biochemistry, chosen to stimulate interest in the subject among students and faculty members, are made available along with a summary discussing their relevance. It also has a discussion board where queries in biochemistry can be posted and these are answered by a member of the faculty. At present, these resources are available only to students and faculty of the Christian Medical College, Vellore, India. However, the institution is working towards making these resources available as open educational resources (OERs) that can be freely accessed.

Several studies have compared the effectiveness of e-resources as learning tools with that of lectures [8-10]. There are, however, very few reports on the use of e-learning as an adjunct to conventional lecture-based teaching. In this regard, Shanthikumar [11] has reported that use of podcasts was effective in enhancing medical students’ learning when provided along with conventional lectures, while Lancaster et al. [12] have made similar observations with the use of technology to provide “blended learning modalities” in a course for nursing students. We carried out this study in an attempt to assess the extent to which medical students would use e-resources, if they were provided. We also wished to determine whether the extent of use of these resources correlated with their performances in formative and summative assessments in the subject. In addition, we aimed to ascertain their perceptions of the impact of the e-resources on their learning in biochemistry and to obtain feedback on and suggestions for improvement of the quality of the e-resources provided.

## Methods

Sixty first-year medical students in Christian Medical College, Vellore, India, were the subjects of this study. They constituted the entire first-year class. Biochemistry is one of the subjects in the first year of the medical course and is taught over a period of a year. Each week’s teaching schedule in the subject consisted of 3–4 hours of lectures and a practical session. All lecture presentations (PowerPoint slides) were made available to first-year medical students on the institutional e-learning website. The additional learning material available on the site has been detailed above.

### Administration of a questionnaire

At the end of the academic year, the students were administered a questionnaire to assess various aspects of their use of the e-learning resources provided by the Department of Biochemistry (see Additional file 1). Students evaluated the overall quality of the learning material available on the e-learning website. They were asked questions regarding the frequency with which they accessed the resources and the extent to which they found it useful in studying biochemistry. In addition, they were asked to indicate the extent to which they used resources available to them (textbooks in the subject and e-resources) while preparing for various assessments in the 14 major topics in biochemistry during the year.

Students were also asked to provide feedback on how access to the e-resources affected their classroom behavior (such as note taking and attentiveness in class) and learning experience (such as the extent to which they understood topics in biochemistry, whether the availability of the material had stimulated their interest in the subject, whether it motivated them to study it, how it affected their ability to perform in various assessments and the extent to which they read textbooks). Students were also asked what their opinion of the new venture was, when they were first introduced to it at the beginning of the academic year and if their opinion had changed by the end of the academic year.

### Analysis of data obtained from the questionnaire

A detailed analysis of the data obtained from the questionnaire was done. To assess the extent to which students used the resources on the e-learning website, a usage score was calculated. A 5-point Likert scale was used for this purpose, where 1 = used textbooks only; 2 = used textbooks predominantly with minimal use of e-resources; 3 = used both textbooks and e-resources equally; 4 = used e-resources predominantly with minimal use of textbooks and 5 = used e-resources only. The usage score for each student was calculated from the sum of the individual scores given by the student for each of the 14 major topics in the course. Students who obtained an overall score less than 28 were designated “low users”, those scoring 29–42 were “moderate users” and those scoring 43 or more, were designated “high users” of the e-resources. The low users studied mainly from text books, while the high users studied mainly from e-resources. The moderate users used both resources to approximately equal extents.

Next, we analyzed data relating to students’ performances in various assessments conducted during the academic year. The marks secured by students in periodic formative assessments during the course and the summative assessment at the end of the course were obtained from departmental records. The average marks obtained by the low, moderate and high users were calculated for all the periodic assessments and the final assessment conducted. Data was analyzed by one way analysis of variance (ANOVA) and Pearson’s correlation coefficient, using Statistical Programme for Social Sciences (SPSS version 16). Bonferroni’s correction was used as the post-hoc test for pair-wise comparisons between the different e-learning usage groups.

## Results

**Figure 1:**
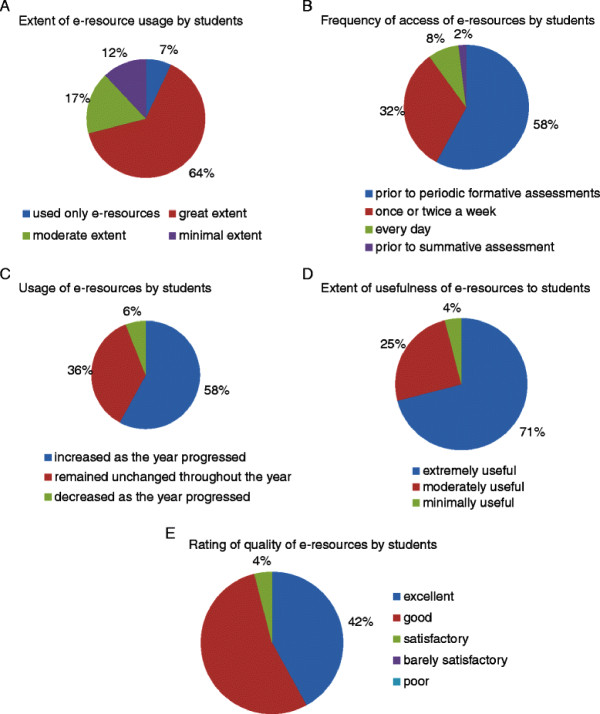
Various aspects of usage of e-resources by students.

The mean age of the subjects in this study was 19.5 ± 0.85 years. They consisted of 34 males (56.67%) and 26 females (43.33%). Almost all the students (59 out of 60) used the e-learning resources to varying extents during the academic year. Among them, a majority of them (64%) said they used the used the e-resources to a “great extent”. A small group of students (7%) said they used this resource exclusively to study biochemistry (Figure 1A, item 3 in questionnaire). When asked about the frequency with which they accessed the e-resources, 58% of them said that they accessed the material just prior to periodic formative assessments, while 32% of students did so once or twice a week on a regular basis (Figure 1B, item 4 in questionnaire). The majority of them (58%) also said that they accessed the e-resources more frequently as the year progressed (Figure 1C, item 7 in questionnaire). Seventy one percent of students found the material available “extremely useful” (Figure 1D, item 5 in questionnaire). Most students rated the overall quality of learning material on the website as excellent (42%) or good (54%) (Figure 1E, item 11 in questionnaire).

**Figure 2:**
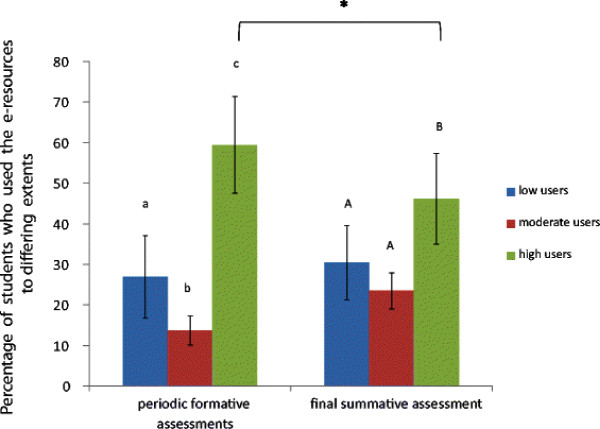
**Percentage of low, moderate and high users of e-resources in biochemistry for periodic formative assessments and final summative assessment.** Students were asked to rate the extent to which they used e-resources to study each of the 14 major topics in biochemistry, both for periodic formative assessments as well as the final summative assessment. Based on their responses, they were classified as low, moderate and high users of the e-resources, as described under “Methods”, for each of the 14 topics. The bars in the figure denote the means of the numbers of users in each category, when data for all the topics were combined. The error bars indicate the standard deviation of the number of students classified as low, moderate and high users of the e-resources for all the topics combined. Data were analyzed by one-way ANOVA, followed by Bonferroni’s correction (for pair-wise comparisons). Bars, within each assessment group, labeled with different letters are significantly different from one another (p < 0.05). * indicates p < 0.05 when comparisons were made within each user group in the formative and summative assessment categories.

The majority of students depended on the e-resources to study various topics in biochemistry for their formative and summative assessments (Figure 2, item 6 in questionnaire). However, students tended to utilize textbooks to a greater extent to prepare for their summative assessment when compared to the periodic formative ones. This is reflected by a statistically significant fall in the average number of students who depended mainly on e-resources (high users) from nearly 60% for the formative assessments to 46.1% for the summative one (p = 0.005). Changes seen in the numbers of moderate and low users were not statistically significant (Figure 2).

**Figure 3:**
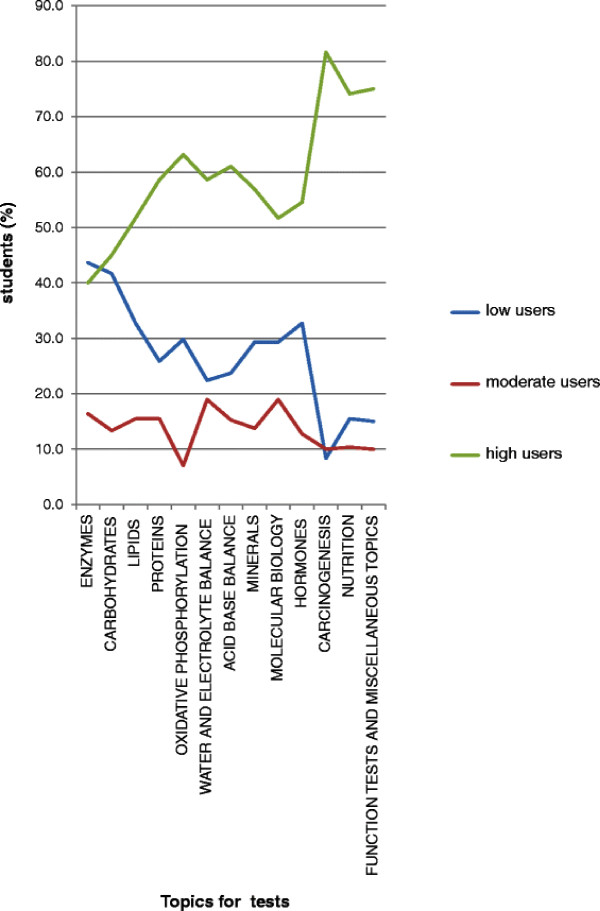
**Trends in the usage of e-resources by students (in the categories of high, moderate and low users) to prepare for periodic formative assessments in various topics in biochemistry during the academic year.** On the x-axis, the topics for periodic formative assessments (tests) are shown in chronological order, with “Enzymes” being the first test and “Function tests and miscellaneous topics” being the last test in the academic year.

We further analyzed the study habits of students during the course of the year (Figure 3). We found that, as the academic year progressed, the number of high users increased from 40% at the start to nearly 80% at the end of the course, with corresponding falls in the number of low users. The number of moderate users at the start of the course (15%) decreased (to about 10%) by the end of the academic year.

**Figure 4:**
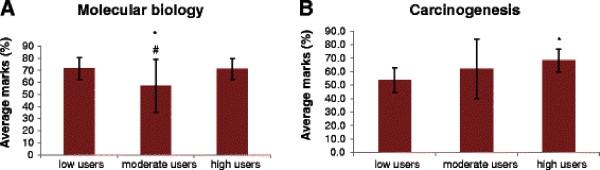
**Average marks obtained by student in the test on molecular biology (A) and carcinogenesis (B).** * indicates p < 0.05 when compared to “low users” and # indicates p < 0.05 when compared to “high users”.

In order to determine whether the extent of use of the e-resources had an effect on students’ performances in the assessments carried out, we compared the marks obtained by different groups of students in the formative and final summative assessments. In the periodic assessments on various topics, there were no significant differences in the marks obtained by low, moderate and high users, except in the tests on molecular biology and carcinogenesis (Figure 4). In the test on molecular biology, post-hoc analysis showed that there was no significant difference between the average marks obtained by the low and high users. However, moderate users scored significantly lower marks than both the low and high users. In the test on carcinogenesis, the high users performed best, with their average marks being significantly higher than those of low users, but not those of moderate users. Analysis of marks obtained in the final summative assessment showed that the high users scored the highest average marks (61.1 ± 9.1%) followed by the moderate (60.5 ± 10.0%) and low users (58.6 ± 7.6%). However, these differences were not statistically significant.

There was a statistically significant relationship among the marks obtained in the formative tests and pre-final assessment (correlation coefficient = 0.75; p < 0.001) and final examination (correlation coefficient = 0.66; p < 0.001), suggesting that students who did well in the formative assessments were able to perform well in the assessments at the end of the course. The relationships between use of e-learning resources and final assessment marks, gender, age and different high school examination boards were not statistically significant.

**Figure 5:**
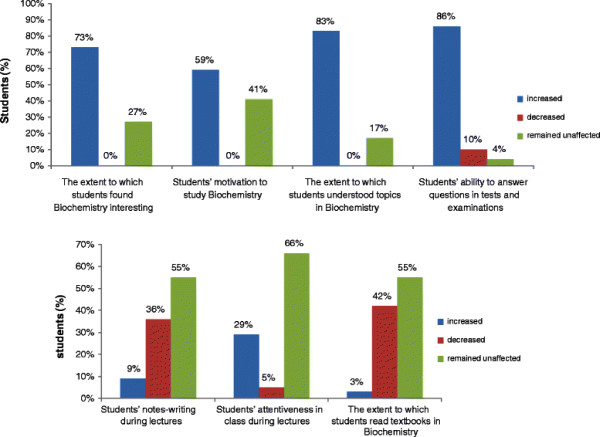
Students’ responses about the impact of e-resources on various aspects of their learning in biochemistry.

Students were also asked to assess the impact of e-learning resources on their study habits and classroom learning. The results obtained from analysis of this data are summarized in Figure 5 (item 8 in questionnaire). It is seen that availability of the e-resources resulted in 36% of students decreasing their note-taking in class and 29% stating that their attentiveness in class had increased. Eighty three percent and 86% said that the extent to which they understood biochemistry and their ability to answer questions in assessments respectively, improved as a result of having access to the e-resources. Seventy three percent and 59% of the students stated that their interest in Biochemistry as a subject and their motivation to study it respectively, increased on account of having the e-resources. Forty two percent of the students said that they read textbooks less as a result of this.

**Figure 6:**
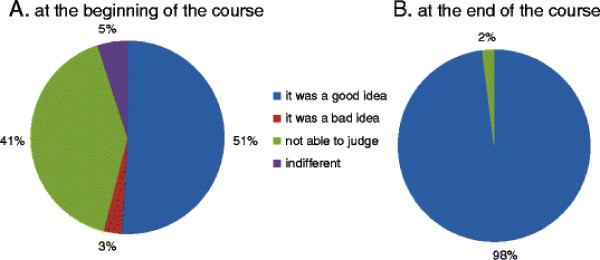
Students’ opinions regarding e-resources at the beginning of the course (A) and at the end of the course (B).

Fifty one percent of students said that they thought it was a “good idea” when they were told about the availability of e-resources at the start of the academic year, 41% were not able to judge, 3% thought it was a bad idea and 5% were indifferent. At the end of the academic year, 98% of students felt that making such resources available for students’ use was a “good idea” and that the department should continue to provide this resource to students in the future (Figure 6, items 14 and 15 in questionnaire).

## Discussion

The results of our study provided several interesting insights. Usually, medical students use textbooks and their classroom notes to prepare for assessments in biochemistry during their first year. The resources on the e-learning website were an additional source of information made available to the students in the current study. Almost all the students used the e-resources that were provided by the department. The majority of students (96%) gave a high rating to the quality of the material provided.

It has been shown that provision of e-learning material in the form of audio-visual podcasts consisting of PowerPoint sides with a voice-over narrative was popular among students as a revision aid prior to assessments [11]. Fifty two percent of students in this study said that they used the e-resources primarily to prepare for the periodic assessments during the course. We found that the number of students who used e-learning resources as their major source (high users) to prepare for the periodic formative assessments increased steadily as the academic year progressed (Figure 3). This was also reflected in the fact that the number of students who primarily depended on text books (low users) declined steadily as the year progressed. This trend may be because of two reasons: firstly, the students may have found that the e-resources provided information that was concise and comprehensive to use to prepare for assessments. It often takes longer to prepare to the same extent using textbooks. On being asked specifically about what they liked about the e-resources (item 12 in questionnaire), some of the responses from students were: *“e-resources were easily accessible, concise, comprehensive and easy to recollect in examinations*”, “*It was helpful to know what the important topics were; it is often not possible to get such info from text books*”. These features obviously made the use of e-resources attractive to students. Secondly, the students may have found that some of the topics were not covered adequately or in an easily comprehensible way in the textbooks they use. Indeed, some students did say that “*there was more information on the e-learning website than in textbooks for some topics*”. This was true of some of the topics towards the end of the academic year (e.g., carcinogenesis). In fact, more than 80% of students were high users of the e-resources for this topic (Figure 3). This may account for the fact that high users of the e-resources scored significantly higher marks in the assessment on carcinogenesis (Figure 4). When asked about their preparation for the final summative assessment, it was found that students used textbooks to a greater extent than they did to prepare for periodic assessments (Figure 2). Nevertheless, we found that e-resources still remained the major source of information for students to prepare for the final summative assessment in many of the topics in biochemistry (Figure 2).

Note-taking during lectures is a useful strategy that many students use for learning purposes, as it keeps them attentive to the lecturer and helps them avoid having to refer to diverse sources to get information required on a particular topic [13,14]. A major disadvantage of this, however, is that it is often difficult to listen to the lecturer and take notes at the same time. This frequently results in students’ lecture notes being patchy and incomplete. If students are assured of access to the lecture presentations outside of class hours (via the e-learning website), they would give their full attention to the lecturer and refer to the presentation later. When asked about this, some of the responses from students were: *“since I could get the lecture material later, I stopped scribbling notes in class and paid more attention to the teacher. This really helped me*”, “*I write very slowly; this helped me just listen to the teacher and prepare my notes later*”. We found that 36% of students said that their note–taking during lectures had decreased, when they knew the presentations would be available on the e-learning website for them to refer to. In addition, 29% of students said that their attentiveness in class increased as they were able to give their full attention to the lecturer. Thus, about a third of the students have benefited in these ways.

Performance of students in assessments depends on the extent of their understanding of the subject and their ability to focus on its most important elements. In this regard, the fact that 86% of students thought that they were better equipped to answer questions in assessments, as a result of access to e-resources, is an important observation. Many students said that “*e-resources helped to quickly revise all the topics prior to exams*” and “*it helped me make sure that I was prepared to answer the most important questions in exams*”. Some studies have shown that e-learning improved performance of students when compared to traditional teaching methods [15-17]. However, we did not find significant differences in the performances of the various usage groups in the final summative examination at the end of the course, when using the marks that they obtained in their test as the outcome. Nevertheless, the fact that being able to access and study from the e-resources gave students a sense of mastery over the subject and gave them confidence to perform is a very positive aspect of this intervention.

We have frequently found that first-year medical students are unable to appreciate the clinical relevance of biochemistry and hence tend to perceive it as being uninteresting. The undue and unwarranted importance placed on rote learning of various biochemical pathways in the subject and failure of teachers to adequately emphasize its clinical relevance contribute a great deal to this situation. In this context, it is extremely encouraging to find that 73% of students in the study said that they found biochemistry interesting. In addition, 86% of students said the e-resources enabled them to understand the subject better and 59% said they felt motivated to study it (Figure 5). Hence, the students’ interest in and understanding of the subject seemed to have improved as a result of access to e-resources. These are extremely encouraging findings and will help in sustaining interest of medical students in biochemistry, as they progress into the clinical phase of the medical course.

While providing access to e-resources appears to have helped first-year medical students in many ways, one has to be aware of possible disadvantages as well. In this study, 42% of students said that their dependence on text books to learn biochemistry decreased as a result of having access to the e-resources (Figure 5). When asked about the aspects of e-learning that students disliked (item 13 in questionnaire), some of the responses were: “*I read textbooks extensively initially but I found that my classmates who studied from the lecture presentations were scoring more marks than I did. So, I started studying from the e-resources too. However, I don’t think that this was good”, “I did not feel the need to read textbooks. The info from the e-resources appeared sufficient to do well in the exams”*. This can be a cause for concern since the habit of reading textbooks is an important aspect of self-directed learning [18]. Dependence on e-resources as the sole source of information on a topic may limit the knowledge acquired by students to the bare essentials required to pass examinations. In order to circumvent this, it is necessary that medical teachers emphasize that e-resources are only a supplemental aid to learning and that frequent student-teacher interactions and textbooks should remain the primary sources of knowledge. In addition, teachers should also design formative and summative assessments in the course such that students are also tested on additional material that is not available in the e-resources and/or on aspects that test the ability of students to apply their knowledge.

## Conclusions

The results presented here show that students perceived that e-learning resources had made a positive impact on various aspects of their learning in biochemistry. The attitude of the majority of students towards learning biochemistry from e-resources was very positive and they overwhelmingly recommended its continuation as part of the course.

## Competing interests

The authors declare that they have no competing interests.

## Author’s contributions

JV and MJ conceived and designed the study, collected, analyzed and interpreted the data, and wrote the manuscript. MF contributed towards acquisition of data and reviewing of the manuscript. All authors have approved the final manuscript that was submitted to the journal.

## Authors’ information

All authors are actively involved in teaching first-year medical students biochemistry at Christian Medical College, Vellore, India. MJ is currently Professor, MF is Associate Professor and JV is Assistant Professor in the Department of Biochemistry.

## Pre-publication history

The pre-publication history for this paper can be accessed here:

http://www.biomedcentral.com/1472-6920/12/21/prepub

## Supplementary Material

Additional file 1 Questionnaire that was used in the study. Click here for file
