# Supplementary material for: Impact of e-resources on learning in biochemistry: first-year medical students’ perceptions
Source: BMC Med Educ. 2012 May 16;12:21. doi: 10.1186/1472-6920-12-21 (PMC3353857; doi:10.1186/1472-6920-12-21)
Supplement: Additional file 1 — Questionnaire that was used in the study. [file 1472-6920-12-21-S1.doc]

**Table 1:** Questionnaire

**1. Did you use the e-learning website to study Biochemistry at any point during your first year?**
i) Yes ii) No

**2. If you answered "no" to question 1, what were the reasons you did not use the material?**

**3.  If you answered "yes" to question 1, to what extent did you use the material during your first year?**
i) Minimally ii) To a moderate extent iii) To a great extent iv) Used only this material

**4. How would you best describe the frequency with which you accessed the e-learning website to access material in Biochemistry?**
i) Every day ii) Once or twice a week on a regular basis
iii) Prior to periodic formative assessments iv) Prior to final summative assessment only.

**5. To what extent did you find the material on the site useful?**
i) Not useful at all ii) Useful to a minimal extent
iii) Useful to a moderate extent iv) Extremely useful

**6. What were your learning resources for studying the following topics in Biochemistry?
Use the following key to answer this question.**
1 = text books only
2 = mainly text books with minimal use of the e-learning website
3 = equally from text books and the e-learning website
4 = mainly from the e-learning website with minimal use of text books
5 = only from the e-learning website

**Topic 1: Enzymes**
a. formative assessment                           1     2     3     4    5    
b. summative assessment   1     2     3     4    5

**Topic 2: Carbohydrate metabolism**
a. formative assessment                           1     2     3     4    5    
b. summative assessment   1     2     3     4    5

**Topic 3: Lipid metabolism**
a. formative assessment                           1     2     3     4    5    
b. summative assessment   1     2     3     4    5

**Topic 4: Protein metabolism**
a. formative assessment                           1     2     3     4    5    
b. summative assessment   1     2     3     4    5

**Topic 5: Molecular biology**
a. formative assessment                           1     2     3     4    5    
b. summative assessment   1     2     3     4    5

**Topic 6: Water and electrolyte balance**
a. formative assessment                           1     2     3     4    5    
b. summative assessment   1     2     3     4    5

**Topic 7: Regulation of pH**
a. formative assessment                           1     2     3     4    5    
b. summative assessment   1     2     3     4    5

**Topic 8: Minerals**
a. formative assessment                           1     2     3     4    5    
b. summative assessment   1     2     3     4    5

**Topic 9:** **Oxidative phosphorylation**
a. formative assessment                           1     2     3     4    5    
b. summative assessment   1     2     3     4    5

**Topic 10: Hormones**
a. formative assessment                           1     2     3     4    5    
b. summative assessment   1     2     3     4    5

**Topic 11: Carcinogenesis**
a. formative assessment                           1     2     3     4    5    
b. summative assessment   1     2     3     4    5

**Topic 12: Nutrition**
a. formative assessment                           1     2     3     4    5    
b. summative assessment   1     2     3     4    5

**Topic 13: Function tests**
a. formative assessment                           1     2     3     4    5    
b. summative assessment   1     2     3     4    5

**Topic 14: Miscellaneous topics**a. formative assessment                           1     2     3     4    5    
b. summative assessment   1     2     3     4    5

**7. Did your usage of the e-learning website**:
i) increase as the year progressed ii) decrease as the year progressed
iii) remained unchanged through the year

**8. Indicate how the availability of learning material on the e-learning website has made an impact on the following aspects of your life as a first year student:**

**i) Your taking notes in class:**
a) increased b) decreased c) was unaffected

**ii) Your level of attentiveness in class**:            
a) increased b) decreased c) was unaffected

**iii) The extent to which you read text books in Biochemistry**a) increased b) decreased c) was unaffected

**iv) The extent to which you have understood topics in Biochemistry**
a) increased b) decreased c) was unaffected

**v) Your ability to answer questions in tests and examinations**
a) increased b) decreased c) was unaffected

**vi) The extent to which you found Biochemistry interesting**
a) increased b) decreased c) was unaffected

**(vii) The motivation you felt to study Biochemistry**
a) increased b) decreased c) was unaffected

**9. How good was access to the e-learning website in terms of the following features?**Use the following key to rate each feature:
1 = poor; 2 = barely satisfactory; 3 = satisfactory; 4 = good; 5 = excellent

i) Time available to access the e-learning website:                 1       2         3        4        5
ii) Availability of computers in the computer centre:   1         2         3        4        5
iii) Internet connectivity and time of download:                       1         2         3        4        5

**10. Should learning material in Biochemistry continue to be made available on the e-learning website?**
i) Yes                                   ii) No

**11. How would you rate the overall quality of the Biochemistry content on the e-learning website?**
i) poor ii) barely satisfactory iii) satisfactory iv) good v) excellent

**12. What are 2 advantages you found in accessing learning material in Biochemistry on the e-learning website?**

**13. What are 2 disadvantages you found in accessing learning material in Biochemistry on the e-learning website?**

**14. At the start of your course when you were first told about the e-learning website, what was your opinion of the new venture? Pick the option below that best describes what you thought.**
i) It was good idea ii) It was a bad idea iii) You were not able to judge
iv) You were indifferent                   v) Any other opinion (please specify)

**15. At the end of your course, what was your opinion about the e-learning website? Pick the option below that best describes what you thought.**
i) It was good idea ii) It was a bad idea iii) You were not able to judge
iv) You were indifferent                   v) Any other opinion (please specify)

**16. Please give any suggestions you have on how the e-learning website can be further utilized for first year medical students to learn biochemistry.**
